# Determinants of breastfeeding initiation within the first hour of life in a Brazilian population: cross-sectional study

**DOI:** 10.1186/1471-2458-10-760

**Published:** 2010-12-09

**Authors:** Tatiana O Vieira, Graciete O Vieira, Elsa Regina J Giugliani, Carlos MC Mendes, Camilla C Martins, Luciana R Silva

**Affiliations:** 1 State University of Feira de Santana, Bahia, Brazil; 2 Federal University of Bahia, Bahia, Brazil; 3 Federal University of Rio Grande do Sul, Rio Grande do Sul, Brazil

## Abstract

**Background:**

Breastfeeding within the first hour of life is a potential mechanism for health promotion. The purpose of this study was to evaluate the prevalence of breastfeeding initiation within the first hour of life in Feira de Santana, Bahia, Brazil, between 2004 and 2005, and investigate the influence of maternal, child and prenatal factors on this practice.

**Methods:**

This is a cross-sectional study extracted from the results of a contemporary cohort conducted in 10 maternity hospitals in the city of Feira de Santana, Bahia, Brazil. A group of 1,309 mother-child pairs was included in the study. Information about mother's and baby's characteristics, pregnancy, birth, and time of breastfeeding initiation was collected in the first 72 hours after delivery, through interview with mothers and hospital records. The data gathered were stored and analyzed using the SPSS 16.0 and R 8.0. The chi-square test and binary logistic regression analysis were used to examine the relationship between breastfeeding within the first hour and different variables.

**Results:**

47.1% of the mothers initiated breastfeeding within the first hour after birth. Early initiation of breastfeeding was associated with birth at full term pregnancy (adjusted Prevalence Ratio 1.43; 95% confidence interval 1.10 to 2.00), mothers who received prenatal guidance regarding the advantages of breastfeeding (aPR1.23; 95% CI 1.11 to 1.41) and vaginal delivery (aPR 2.78; 95% CI 2.38 to 3.23).

**Conclusions:**

In order to improve the rates of breastfeeding within the first hour of life, health care professionals must promote the factors favoring this practice such as prenatal guidance regarding the advantages of breastfeeding, vaginal delivery and full term birth, and stimulate this practice in vulnerable situations such as mothers with cesarean section and preterm birth.

## Background

Within the search for mechanisms and actions to reverse the trend towards early weaning, measures have been taken aimed at promoting, encouraging and supporting breastfeeding. Among these measures, the following can be highlighted: modifications of prenatal routines in maternity hospitals; greater value placed on breastfeeding within the first hour of life, in the delivery room; rooming-in; and measures after discharge from hospital [1].

Although breastfeeding within the first hour of life is considered to be an indicator of excellence of breastfeeding [2], the characteristics associated with this practice have been little investigated. Nonetheless, certain facilitating factors have already been described: skin-to-skin contact between mother and baby immediately after birth [3], being a young mother, lower maternal schooling levels, lower family income and vaginal delivery [4]. On the other hand, characteristics such as previous experience with breastfeeding and prenatal knowledge of the advantages of breastfeeding require further investigation.

A study conducted in 2001 in the city of Feira de Santana, Brazil, demonstrated that the prevalence of breastfeeding initiation within the first hour of life was 52.2%. However, the study did not state the factors associated with this practice [5].

Within this context, the purpose of the present study was to investigate the prevalence of breastfeeding initiation within the first hour of life in Feira de Santana, Brazil, between 2004 and 2005, and investigate the influence of maternal, child and prenatal factors on this practice. Another aim of the study was to investigate the prevalence of breastfeeding within the first hour of life, in relation to mother, child and prenatal care characteristics.

## Method

### Study Design

This is a cross-sectional study extracted from the results of a contemporary cohort of live births conducted in all 10 public and private maternity hospitals in the city of Feira de Santana, Bahia, Brazil.

### Sample size

All mothers delivering in any hospital in the city of Feira de Santana between 2004 and 2005 and their babies were eligible for the study. In a similar study conducted in Brazil in 2003, 35% of children were breastfed in the first hour after delivery [4]. It was estimated that there would be around 10,177 deliveries during 2004. We estimated the sample size for this project using the following parameters: 95% for the confidence level; 10,177 for the population size; 0.35 for the estimated true proportion; and +/- 0.03 as Confidence limits. Thus, the sample size required was 887. Data from 1,309 mother-child pairs were gathered.

### Variables

Information about mother's and baby's characteristics, pregnancy, birth, and time of breastfeeding initiation was collected in the first 72 hours after delivery, through interview with mothers and hospital records. The following data were collected: birth weight (low birth weight was considered to be <2,500 g) and gestational age at birth (premature if <37 weeks); mother's age on delivery (young mother was considered to be <20 years), educational level; parity (first-time mother or multiparity); previous experience with breastfeeding; prenatal breastfeeding class; prenatal guidance regarding the advantages of breastfeeding (not necessarily advantages of breastfeeding within the first hour of life); monthly family income (presented as unit of U.S. dollars, equivalent to two Brazilian minimum wage, classified as less than or greater than US$346.80); method of delivery (vaginal or cesarean/forceps delivery) and the timing of the breastfeeding initiation, according to mother information, which was expressed as a categorical variable using the first hour after birth as the cutoff point.

### Statistical Analysis

This study used the data from the questionnaires applied in the hospital. Data were typed twice and EpiData Entry software was used for validation of the typing. The data were analyzed using the Statistical Package for the Social Sciences for Windows version 16.0 software (Chigago, II, USA) and the R 8.0 statistical software.

Data descriptions and analyses were based on the outcome sample of 1,309 mother/child pairs. The sample was described using frequencies. On bivariate analysis, the measure of association used was the prevalence ratio with a 95% confidence interval. Then, multivariate analysis were done through logistic regression. Initially, the association of each independent variable with the dependent variable was tested through logistic regression and those with a *p *value of 25% or lower passed to the second stage, then a model was constructed with the variables selected on the previous stage using backward selection and a level of significance of 0.17. On the final stage, the variables kept on the second stage were placed together on the model, using the backward selection and a level of significance of 0.05, thus determining the logistic regression coefficients, prevalence ratio and their 95% confidence intervals. The fit of the model was verified with the Hosmer-Lameshow test and residual analysis was also performed. A generalized linear model with a logarithmic link function and binomial distribution for the residual (GLM log-binomial) was used. The method provides a good approximation of variance estimates [6,7].

The research protocol observed the rules on research involving human subjects (CNS Resolution 196/96) [8], it was in compliance with the Helsinki Declaration, and it was approved by the Human Subjects Ethics Committee (institutional review board) of the State University of Feira de Santana, Bahia, Brazil (N° 092/2008). All women gave informed consent.

## Results

### Study population and prevalence of breastfeeding initiation within the first hour of life

Forty-seven percent (616/1,309) of the newborns were breastfed within one hour from birth. Among the infants, 46.7% were female (611/1,309), 1,246 had a birth weight greater than 2,500 g (95.2%) and 4.8% were premature (63/1,309).

Among the mothers, about half was first-time mothers (50.2%); 48.3% had previous experience with breastfeeding; 19.3% were less than 20 years old; and only 1,262/1,309 answered about prenatal breastfeeding class. The others characteristics are presented in the Table 1.

**Table 1:** Description of socio-economic, demographic and pre-natal characteristics.

Variables	N	%
**Prenatal breastfeeding class (1,262)**		
Yes	337	26.7
No	925	73.3
**Maternal educational level (1,309)**		
>4 years of study	816	62.3
≤4 years of study	493	37.7
**Montly family income (1,309)**		
≥2 minimum wage (greater than US$346.80)	604	46.1
<2 minimum wage (less than US$346.80)	705	53.9
**Prenatal guidance regarding advantages of breastfeeding (1,309)**		
Yes	829	63.3
No	480	36.7
**Method of delivery (1,309)**		
Vaginal	731	55.8
Cesarean or forceps	578	44.2

### Influence of the characteristics of the mother, child and prenatal care

Bivariate analysis showed a significantly lower rate of breastfeeding in the first hour after delivery among mothers older than 20 years, with higher educational level and monthly family income bigger than two minimum wage. The women who were more likely to initiate breastfeeding during the first hour after birth had prenatal guidance regarding the advantages of breastfeeding and had vaginal delivery (Table 2).

**Table 2:** Chi-square bivariate analysis on characteristics associated with breastfeeding initiation in the first hour after birth

Variables	Breastfeeding within the first hour of life		Prevalence Ratio (95% Confidence Interval)	p
			
	Yes (%)	No (%)		
**Birth weight**				
≥2,500 g	593 (47.6)	653 (52.4)	1.30 (0.94-1.82)	0.085
<2,500 g	23 (36.5)	40 (63.5)		
**Gestational age at birth**				
Term	592(47.5)	654 (52.5)	1.25 (0.91-1.72)	0.144
Preterm	24 (38.1)	39 (61.9)		
**Parity**				
Multiparity	319 (48.9)	333 (51.1)	1.08 (0.97-1.21)	0.180
First-time mother	297 (45.2)	360 (54.8)		
**Mother's previous experience with breastfeeding**				
Present	309 (48.9)	323 (51.1)	1.08 (0.96-1.21)	0.199
Absent	307 (45.3)	370 (54.7)		
**Mother's age at delivery**				
≥20 years	477 (45.2)	579 (54.8)	0.82 (0.72-0.94)	0.005
<20 years	139 (54.9)	114 (45.1)		
**Maternal educational level**				
>4 years of study	360 (44.1)	456 (55.9)	0.85 (0.76-0.95)	0.006
≤4 years of study	256 (51.9)	237 (48.1)		
**Montly Family income**				
≥2 minimum wage	228 (37.7)	376 (62.3)	0.69(0.61-0.78)	<0.001
<2 minimum wage	388 (55.0)	317 (45.0)		
**Prenatal breastfeeding class**				
Yes	171 (50.7)	166 (49.3)	1.11 (0.98-1.26)	0.116
No	445 (45.8)	527 (54.2)		
**Prenatal guidance regarding the advantages of breastfeeding**				
Yes	437 (52.7)	392 (47.3)	1.41 (1.24-1.61)	0.000
No	179 (37.3)	301 (62.7)		
**Method of delivery**				
Vaginal	481 (65.8)	250 (34.2)	2.82 (2.41-3.30)	<0.001
Cesarean or forceps	135 (23.4)	443 (76.6)		

The results of the multivariate analysis revealed three factors associated with breastfeeding initiation within the first hour of life: mothers who received prenatal guidance regarding the advantages of breastfeeding; vaginal delivery; and full term pregnancy (Table 3).

**Table 3:** Binary logistic regression analysis on factors associated with breastfeeding initiation in the first hour after birth.

Covariables	Adjusted prevalence ratio	95% confidence interval
**Prenatal guidance regarding the advantages of breastfeeding**		
Yes	1.23	1.11-1.41
No	1.0	
**Method of delivery**		
Vaginal	2.78	2.38-3.23
Cesarean/forceps	1.0	
**Gestational age at birth**		
Term	1.43	1.10-2.00
Preterm	1.0	

## Discussion

### Study population and prevalence of breastfeeding initiation within the first hour of life

The prevalence of breastfeeding initiation within the first hour of life in Feira de Santana, Bahia, Brazil in this study was 47.1%. This is considered "low" according to the classification of the World Health Organization. This prevalence was lower than the values published in a recent survey about breastfeeding prevalence, conducted in the Brazilian state capitals and the Federal District by the Ministry of Health, which showed a rate of breastfeeding within the first hour of life of 67.7% [2]. It was also lower than the percentage found in the survey carried out in Feira de Santana in 2001 (52.2%), in which the same methodology as in the Ministry of Health survey was used, i.e. a cross-sectional design [5].

However, it is important to note that the data from the present and previous studies are not perfectly comparable, since they were obtained using different methodologies. The present study was a cross-sectional study extracted from the results of a cohort of live births in which the information on breastfeeding was gathered directly from the mothers within the first 72 hours after delivery. In the cross-sectional study carried out by the Ministry of Health the information on time of breastfeeding initiation was gathered at an interview with mothers of children less than one year of age, 71.3% of them having more than three months old [2]. This implies a possible recall bias, which could have overestimated the results. In addition, in Brazil a series of governmental and non-governmental initiatives aimed at promoting breastfeeding within the first hour of life has been implemented, including many activities in the 2007 World Breastfeeding Week, which central topic was promotion of early initiation of breastfeeding This would certainly have generated positive attitudes and activities that may have contributed towards increased levels of this practice over the last few years. It is worth emphasizing that the children in the present survey were born in the years 2004 and 2005.

In this sense, the percentage of breastfeeding within the first hour of life in Feira de Santana was greater than in other studies with similar recall methods that were conducted before the 2007 World Breastfeeding Week. For example, in a study conducted in Rio de Janeiro between 1999 and 2001, only 22.4% of babies born through a vaginal delivery were breastfed within the first hour after birth [9]. In another study, in Pelotas, with births between 2002 and 2003, it was found that 35.5% of the mothers initiated breastfeeding within the first hour after delivery [4].

It is also important to draw attention to conceptual differences that, in turn, could have interfered with the results. The prevailing recommendation at the time when the data for the present study were gathered focused on breastfeeding within the first half hour of life, while still in the delivery room [10]. However, from 2006 onwards, the concept was expanded towards giving more value to skin-to-skin contact between newborns and their mothers immediately after birth, for at least an hour or for as long as the mother wished. During this time, mothers are encouraged to recognize the first signs that her baby was ready for breastfeeding [11].

### Influence of the characteristics of the mother, child and prenatal care

In this study, three factors were identified as predictors for breastfeeding within the first hour of life: mothers who received prenatal guidance regarding the advantages of breastfeeding; vaginal delivery; and full term pregnancy.

With regard to the positive association between prenatal education with guidance on the benefits of breastfeeding and greater prevalence of breastfeeding initiation within the first hour of life, no other studies focusing specifically on the first hour of life were identified. However, divergent results relating to prenatal education and the initiation of breastfeeding have been presented in different studies. A randomized controlled trial carried out by MacArthur et al. [12], in Birmingham showed that guidance and information on the advantages of breastfeeding in prenatal follow-up clinics among a needy population of various ethnicities with at least three contacts at different stages of pregnancy, were ineffective for increasing the rate of breastfeeding initiation. On the other hand, Fairbank et al [13] indicated that implementation of pre- and postnatal support programs, along with prenatal education programs among low-income women, either individually or in small groups, had a positive effect on initiation of breastfeeding. World Health Organization and the United Nations Children's Fund have emphasized that it is important to inform pregnant women about the advantages of breastfeeding during the prenatal period, so that they can make a decision based on facts regarding how to feed their children [11].

Another risk factor for delaying the first breastfeeding identified in the present study was delivering by a cesarean section. Several studies have confirmed that even with hospital practices with norms and routines favoring breastfeeding, birth by cesarean section is a significant barrier that inhibits breastfeeding within the first hour of life [9,14]. The first point to consider in this respect is the limitation on the mother's ability to touch her baby, if her arms have been restrained during the surgical procedure. Another point is the analgesia for the mother, which may cause disorganized behavior in the newborn and may result in delay and impairment of the first breastfeeding [15]. Children born from deliveries with analgesia suffer interference with regard to spontaneous seeking of the mother's breast after birth. This is shown through lower frequency of finger and hand movements, less touching of the nipple and breast (licking and sucking) and more crying than observed among children whose mothers did not receive analgesia [16,17]. Even small doses of narcotic analgesics, when administered between three and one hours before delivery, may impair breastfeeding for hours or days [18].

A study in a Chinese society, with high rates of cesarean deliveries, showed that this was a risk factor for weaning in the first and third months of life, whereas there was an inverse outcome among children who were breastfed within the first 30 minutes after birth [19]. Brazilian studies have shown that cesarean birth in the city of Pelotas was associated with twice as much risk of non-breastfeeding within the first hour of life [4]. A study carried out in Rio de Janeiro found that visual or physical contact between mother and baby, along with breastfeeding in the delivery room, was less frequent among women who underwent cesarean delivery, and this was reflected in longer time intervals between delivery and the first breastfeeding [9]. Consequently, additional support is needed for women who undergo cesarean delivery, in order to help them to start breastfeeding as early as possible [20].

As expected, in the present study premature newborns were less breastfed within the first hour of life than did those born at full term. A cohort study in Pelotas, Brazil, in 2004, showed that 10.8% of newborns were late premature births and, in comparison with the full-term newborns, they were at greater risk of not being breastfed within the first hour after birth [21]. Likewise, a study in Japan [22] and another in Massachusetts [23] reported that premature newborns were less likely to receive maternal milk.

It is worth commenting that, depending on birth weight, premature children have peculiarities and specific characteristics related to their own immaturity. These limit the abilities needed for breastfeeding within the first hour of life, such as good coordination of the suction-deglutition-respiration cycle and the breast-seeking reflex [24]. Furthermore, premature newborns spend less time awake and their mothers may have some difficulty in recognizing the signs of hunger.

## Conclusions

Intervention measures are needed to increase the prevalence of breastfeeding initiation within the first hour of life. These measures should be started during the prenatal period, with the development educational actions that place value on and clarify the advantages of breastfeeding within the first hour of life, thereby arousing the willingness and good intentions among mothers, with regard to placing the baby on the breast immediately after birth. Willingness among healthcare professionals is also needed, with regard to supporting and helping mothers in situations of vulnerability that cause delays in the first breastfeeding, such as occurrences of cesarean delivery and prematurity.

## Competing interests

The authors declare that they have no competing interests.

## Authors' contributions

TOV conceived the current study hypothesis, performed the analysis and drafted the paper. GOV conceived the current study hypothesis, designed and conducted the Cohort. ERJG provided statistical guidance and assisted with study design. CMCM provided statistical guidance and contributed in writing the manuscript. CCM contributed in the analysis and writing the manuscript. LRS assisted with study design, supervised the conduct, analysis and the writing of the manuscript. All authors helped to interpret the findings, reviewed and approved the final draft.

## Source of funding

This work was supported by grants from the Research Support Foundation of the State of Bahia (FAPESB) and from the Coordination Office for University-level Personnel Advancement (CAPES).

## Pre-publication history

The pre-publication history for this paper can be accessed here:

http://www.biomedcentral.com/1471-2458/10/760/prepub
